# Psychological burden in patients with sellar masses under conservative and surgical management

**DOI:** 10.1007/s10143-025-03240-7

**Published:** 2025-01-30

**Authors:** Darius Kalasauskas, Andreas Ernst, Sydney Mireri, Naureen Keric, Santhosh G. Thavarajasingam, Wael Omran, Christian Wüster, Florian Ringel, Jens Conrad

**Affiliations:** 1 Department of Neurosurgery, University Medical Centre, Johannes Gutenberg University, Mainz, Germany; 2 Endocrinological Medical Office, Dr. Wael Omran, Mainz, Germany; 3 Medical Center for Hormones & Metabolism, Mainz, Germany

**Keywords:** Sellar mass, Pituitary adenoma, Quality of life, Psychological distress, Anxiety, Depression

## Abstract

The aim of this study was to investigate the level of distress and the quality of life of operated and non-operated patients with pituitary tumors. Patients who presented to a neurosurgical center and two endocrinological services for outpatient follow-up after surgical treatment, as well as those under medical therapy or radiological follow-up without treatment, were invited to participate in the study. Sociodemographic, health-related quality of life and clinical data were assessed. Psychosocial factors were measured using the Distress Thermometer (DT), the Hospital Anxiety and Depression Scale (HADS), the Short Form (SF-36), and the Sino-nasal outcome test (SNOT). Thirty-two postoperative patients and thirty conservatively managed patients (*n* = 21 under medical treatment, *n* = 9 watch and wait), mean age 53, SD 19; 56% female participated in the study. Prolactinomas (35%) and non-functioning pituitary adenomas (21%) were the most common findings. There were no significant differences between conservative and operative groups in mean DT score (4.7 (SD 2.45) vs. 4.9 (SD 3.0), *p* = 0.61), HADS anxiety score (6.4 (SD 3.9) vs. 6.0 (SD 4.3), *p* = 0.76) or depression score (5.7 (SD 4.8) vs. 4.6 (SD 3.6), *p* = 0.50). For patients with ACTH-producing adenomas, the mean anxiety score was significantly higher (10.3 (SD 1.9) vs. 5.9 (SD 4.0), *p* = 0.03). The SNOT score correlated significantly with the DT, HADS-A, and HADS-D scores and therefore was associated with higher psychological distress. The level of self-reported distress in patients with sellar processes was not associated with a specific treatment strategy. ACTH-producing adenomas and manifest nasal symptoms were associated with higher psychological distress.

## Introduction

Sellar masses can originate from a range of sources, including neoplasms, such as pituitary adenoma, craniopharyngioma, and meningioma, to congenital non-neoplastic lesions, such as Rathke cleft cysts. Sellar masses are not uncommon. Pituitary adenomas are the third-most common type of intracranial neoplasms, after meningiomas and gliomas [25]. Moreover, asymptomatic pituitary macroadenomas were found in 0.3% of healthy individuals undergoing high-resolution MRI [36], autopsy findings report incidental pituitary lesions in over 10% of cases [33]. Due to better diagnostic methods, the incidence of sellar processes has increased in recent years [11]. Even though some sellar tumors cause noticeable symptoms (e.g., Cushing disease or visual field defects), others are diagnosed incidentally and remain asymptomatic. The diagnosis of a CNS tumor can cause considerable distress regardless of tumor entity [15]. Symptoms and active treatment, such as surgery, may affect the patient’s socioeconomic situation and ability to work. Undergoing an MRI caused distress in almost 30% of healthy participants who received a whole-body scan [32]. Waiting for the results can also cause significant distress [32]. Moreover, potentially serious incidental findings might require a potentially stressful follow-up [29].

Patients diagnosed with a sellar masses usually are managed either surgically (e.g., large pituitary adenoma, hormone-active non-prolactinoma), treated with medications (e.g., prolactinoma), or through follow-up and watchful waiting (e.g., small or unspecific lesion as an incidental finding). Patients with pituitary tumors report impaired quality of life on both physical and mental measures compared to general population [19], with quality of life generally worse in those with ACTH- and growth hormone-secreting tumors [39]. Patients with these tumors may have a lower quality of life despite normal hormonal levels and the absence of neurological deficits [1], but it can improve after surgical treatment [27], suggesting complex biopsychosocial issues. Moreover, impulse control disorders are well documented in patients with prolactinoma receiving dopamine agonists [6]. However, data on the degree of distress among this patient cohort is lacking, particularly for patients with incidental findings.

The aim of this study was to evaluate psychological burden in patients with sellar masses and to compare the symptoms between patients treated conservatively and surgically.

## Materials and methods

### Study design and patients

Patients for this cross-sectional study were recruited consecutively between 2020 and 2023 at the outpatient service of a neurosurgical department in a university hospital and non-consecutively at two outpatient endocrinological services in Mainz, Germany. Patients were recruited during their routine outpatient visits; approximate initial response rate was 70%. The inclusion criteria were (1) age ≥ 18 years, (2) ability to provide informed consent, (3) radiological diagnosis of sellar mass, and (4) consent to participate. For surgically treated patients, diagnosis was confirmed by histologic examination. For patients with functioning pituitary adenomas treated with medication (e.g., prolactinomas, STH secreting adenomas), the diagnosis was confirmed through endocrinologic examination. In conservatively managed cases, prolactinoma was diagnosed based on prolactin levels exceeding 200 ng/ml and the presence of sellar mass. In certain cases (e.g., suspected Rathke’s cleft cyst, small non-functioning pituitary adenoma), no confirmatory tests were possible under radiological control. Patients with histologically confirmed malignant tumors and suspected malignant sellar tumors were excluded from the study. To assess the impact of surgery on psychological burden, patients were divided into two groups: medical treatment/watch-and-wait group and postoperative group. Patients with significant postoperative deficits, such as bitemporal hemianopsia, and those incapable of self-care (Eastern Cooperative Oncology Group Performance Status ≥ 3) were excluded from the study.

To control for score variation over time, patients were asked to complete the same questionnaires 3 to 6 months after the first examination. The questionnaires were sent to the patients by mail.

### Investigations

After signing a consent form, demographic and tumor-specific data were collected using a questionnaire as well as medical and radiological records. The demographic data included sex, age, level of education (higher than secondary or other), occupation, marital status, and comorbidities. Tumor type, localization, extrasellar extent, size, growth, and current medical treatment were recorded. The time since tumor diagnosis and the last significant tumor-specific event (i.e., tumor diagnosis in patients who received medical treatment and radiological follow-up, surgery, or radiotherapy) were considered.

### Applied questionnaires

Psychosocial factors were measured using German adaptation of the Distress Thermometer (DT), Hospital Anxiety and Depression Scale (HADS), the Short Form (SF-36), and Sino-Nasal Outcome Test (SNOT).

The SF-36 [2, 38] questionnaire is a widely used multidimensional questionnaire measuring health-related quality of life (HRQOL) and consists of 36 items. The outcomes are measured in 8 subscales describing vitality, physical functioning, physical pain, general health perceptions, physical role functioning, emotional role functioning, social role functioning, and mental health. These subscales can be summarized into a physical component score (PCS) and mental component score (MCS).

The HADS questionnaire consists of items measuring depressive and anxiety symptoms, based on 14 questions [14, 40]. The questions assess anxiety and depression and provide respective scores: anxiety score (HADS-A) and depression score (HADS-D). A score of less than 8 was considered normal, 8–10 as mild, and > 10 as manifest.

The DT is a short screening questionnaire assessing psychological burden on a numerical analog scale from 0 to 10 [12]. It is accompanied by a 40-item problem list of emotional, practical, physical, and spiritual concerns. In our study, ≥ 6 on DT scale was considered a significant burden.

SNOT is a 22-item questionnaire developed to evaluate disease-specific HRQOL in chronic rhinosinusitis [18, 30]. It has been validated and widely used to assess postoperative quality of life in patients undergoing pituitary surgery [31]. The SNOT-22 score ranges from 0 to 110, with higher scores indicating more symptoms. A stratification of the score into mild (8–20), moderate (21–50), and severe (> 50) has been proposed [34].

### Statistics

The sample size was calculated based on the primary outcome, the difference in psychological distress between the conservatively and surgically treated patient groups, as measured using the HADS scale. The null hypothesis was no difference in HADS anxiety score between the groups. The sample size was estimated at 31 patients per group to detect a clinically relevant difference of 3 if the standard deviation does not exceed 4, with the maximum possibility of type I error = 5% and type II error = 20%.

Categorical data were described as absolute and relative frequencies and continuous data as means and standard deviations. The secondary outcome was psychological distress, as measured using the SF-36, DT, and SNOT questionnaires. Descriptive analysis was conducted. Differences between the groups were assessed using the chi-squared test, Student t-test, or Mann-Whitney test for independent variables and paired samples t-test or Wilcoxon signed rank test for related variables, as appropriate. Correlation between scores of questionnaires was measured using Spearman correlation. Univariate and multivariate logistic regression analyses were conducted to evaluate the association of clinical and demographic characteristics with the scores on the instruments used. Correction for multiple testing was not performed. *P* < 0.05 indicated statistical significance.

## Results

### Patients

Sixty-two patients, with a mean age of 52.7 (SD 19) years, 56.5% (*n* = 35) female were enrolled in the study. Of these, *n* = 32 were in the operative group and *n* = 30 in the conservative group. The difference arose because one patient was misclassified as being treated conservatively during the recruitment. Prolactinomas were identified in 35%, non-functioning pituitary adenomas in 21.0%, and ACTH-producing adenomas in 6.5% of cases. In 14.5% of cases, the mass entity was unknown, and these patients were followed up with serial MRIs (Table 1). The mean time since the diagnosis was 54.2 (SD 72, range 0.7–340) months, and longer in the postoperative group. The mean time since the last major event (surgery or radiotherapy (*n* = 4)) was 36.4 months (SD 33, range 0-143 months). None of the patients in conservative group underwent radiotherapy.

**Table 1:** Patient and tumor characteristics

		Postoperative	Medical treatment/ Watch and wait	Total	*p*-value
**N**		32	30	62	
**Age (SD)**		56 (15)	49(20)	53(19)	0.17
**Female**,** n(%)**		21(66)	14(47)	35(56)	0.20
**Family situation**,** n(%)**					0.30
Living with partner		16(50)	11(37)	27(44)	
Living alone		9(25)	7(23)	16(26)	
Undisclosed		7(22)	12(40)	19(31)	
**Depression or known malignancy**,** n(%)**		3	2		
Watch and wait*,** n(%)**				9(15)	
**Medical treatment**,** n(%)**				21(34)	
Dopamine agonists, n(%)		1(3)	19(63)	20(32)	
Lanreotide, n(%)			1(3)	1(2)	
**Post-radiotherapy**,** n(%)**		4(13)			
**Pituitary insufficiency**,** n(%)**					
Corticotropic / hydrocortisone supplementation		16(50)	3(10)	19(31)	**0.001**
Thyreotropic insufficiency		13(41)	7(23)	20(32)	0.15
Gonadotropic insufficiency		10(31)	8(27)	18(29)	0.69
Somatotropic insufficiency		1(3)	2(7)	3(5)	0.52
Combined		9(28)	10(33)	19(31)	0.66
**Tumor size**					
Microadenoma, n(%)		3(9)	9(30)	12(19)	0.19
Expansive growth, n(%)		18(56)	13(43)	31(50)	0.45
Progress, n(%)		3(9)	1(3)	4(6)	
Regression, n(%)		0	6(20)	6(10)	
**Tumor entity**					**< 0.001**
Inactive adenoma, n(%)		13(41)		13(21)	
Prolactinoma, n(%)		3(9)	19(63)	22(35)	
ACTH producing, n(%)		4(13)		4(6)	
STH producing, n(%)		2(6)	1(3)	3(5)	
Gonadotroph, n(%)		3(9)		3(5)	
Mixed, n(%)		2(6)		2(3)	
Other pituitary, n(%)		2(6)		2(3)	
Colloid cyst, n(%)		3(9)		3(5)	
Unknown†, n(%)		0	9(30)	9(15)	
**Time after diagnosis**,** months (SD)**		77(87)	29(36)	54(72)	**0.01**
**Time after operation**,** months (SD)**		36(33)		36(33)	

*without hormone substitution, †no definite histological/endocrinological diagnosis possible (radiologically suspected small inactive pituitary adenomas, Rathke cleft cysts)

### Psychological distress as measured using the HADS and DT

**Fig. 1:**
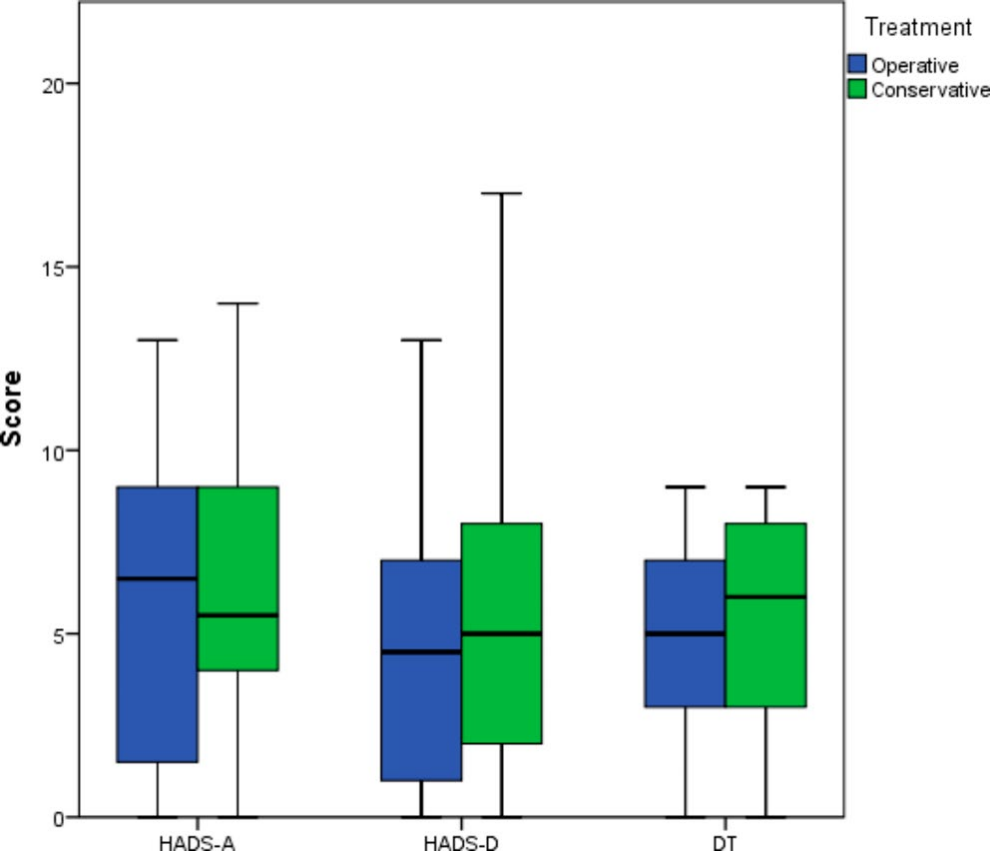
HADS and DT scores across study groups. HADS-A– anxiety score, HADS-D– depression, DT– distress thermometer

The mean anxiety and depression scores for the study population were 6.2 (SD 4.1) and 5.1 (SD 4.2), respectively. There were no significant differences between the conservative and operative groups in mean anxiety score (6.4 (SD 3.9) vs. 6.0 (SD 4.3), *p* = 0.76) or mean depression score (5.7 (SD 4.8) vs. 4.6 (SD 3.6), *p* = 0.50) (Fig. 1). The proportion of patients with high levels of anxiety symptoms was similar in the two groups (21.4% vs. 15.6%, *p* = 0.82). The HADS-A and HADS-D values observed in this study were not markedly different from the normative values in the German population (4.4 and 4.8 for males and 5.0 and 4.7 for females, respectively) [17]. The proportion of patients with high-level depression symptoms was higher in the conservative group (24.1% vs. 9.4%), though this difference did not reach statistical significance (*p* = 0.30). For patients with ACTH-producing adenomas, the mean HADS-A score was significantly higher than for patients without ACTH-producing adenomas (10.3 (SD 1.9) vs. 5.9 (SD 4.0), *p* = 0.03). The HADS-D score was marginally insignificant (8.5 (SD 3.1) vs. 4.8 (SD 4.2), *p* = 0.06).

The mean DT score for study participants was 4.8 (SD 2.7), and the proportion of patients with significant distress was 41.9%. There was no difference between the study groups (4.7 (SD 2.45) vs. 4.9 (SD 3.0), *p* = 0.61) (Fig. 1). The HADS-A, HADS-D and DT scores for different tumor entities are provided in Table 2; statistical comparisons were not performed due to the low number of patients. The most common problems according to the DT problem list were sleep disturbance (55.7%), followed by fatigue (54.1%), pain, and worry (both 52.5%). There was a correlation between DT and MCS (Spearman’s Rho (ρ) -0.50, *p* < 0.001), PCS (ρ -0.40, *p* = 0.002), HADS-A (ρ 0.60, *p* < 0.001), HADS-D (ρ 0.78, *p* < 0.001), and SNOT total score (ρ 0.33, *p* = 0.036).

**Table 2:** HADS and DT scores in different tumor entities at 0 months

Tumor entity	HADS-A, mean (SD)	HADS-D, mean (SD)	DT, mean (SD)
Inactive adenoma			
Prolactinoma	6.4 (3.8)	5.9 (5.1)	4.5 (3.0)
ACTH producing	10.3 (1.9)	8.5 (3.1)	6.0 (2.6)
STH producing	5.3 (5.1)	5.7 (1.5)	6.3 (2.1)
Gonadotroph	9.0 (6.9)	7.7 (3.5)	7.3 (1.2)
Other pituitary	4.3 (4.4)	5.9 (5.1)	4.5 (3.0)
Colloid cyst	5.0 (4.5)	5.9 (5.1)	4.5 (3.0)
Unknown	6.6 (3.9)	4.6 (3.5)	5.9 (2.7)

*due to low number of cases, no statistical comparison between the subgroups was performed

### Quality of life as measured using SF-36

**Fig. 2:**
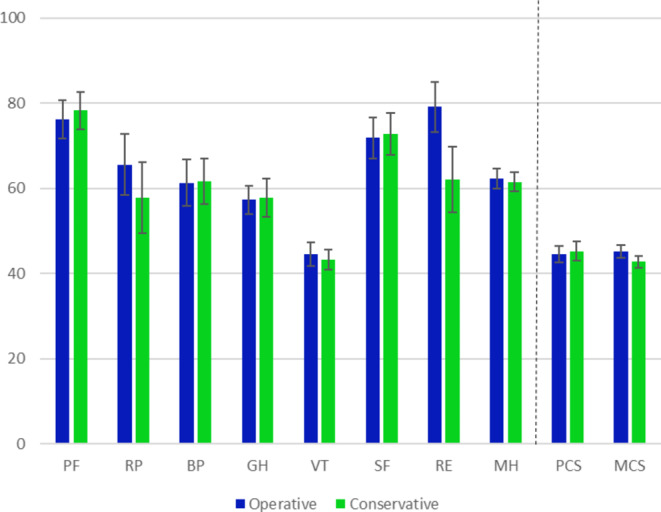
Average value of SF-36 subscales and composite scores in conservatively and operatively managed patients. PF– physical functioning, RP– role physical, BP– bodily pain, GH– general health, VT– vitality, SF– social functioning, RE– role emotional, MH mental health, PCS– physical component summary, MCS– mental component summary

SF-36 analysis demonstrated comparable scores between the conservative and operative groups, with slightly more role limitations due to emotional problems in the conservative group (62.1 (SD 41.5) vs. 79.2 (SD 33.6), *p* = 0.08). In general, self-reported mental and physical health was good, with mean MCS scores of 45.2 (SD 8.7) in the operative group and 42.7 (SD 7.7) in conservative group. The mean PCS scores were 44.6 (SD 10.6) and 45.3 (SD 12.1), respectively (Fig. 2). Since MCS and PCS scores are based on a mean of 50 as mean and a standard deviation of 10 in a representative population sample [6], no marked difference in comparison to general population was identified.

### Sinonasal symptoms

**Fig. 3:**
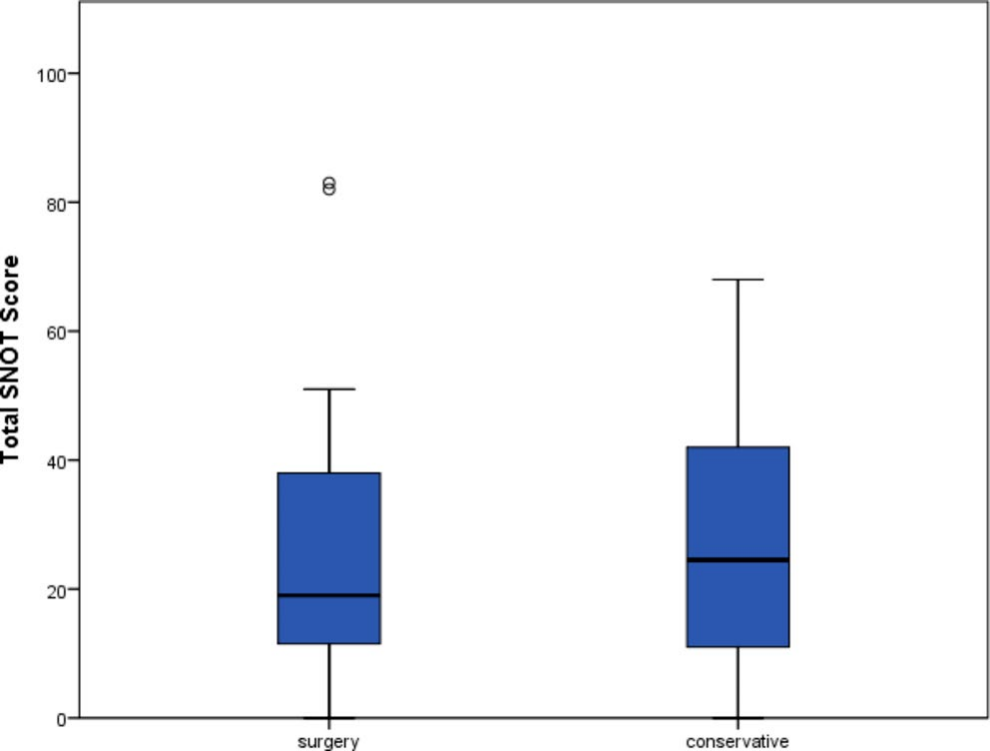
SNOT score in operated and medically treated/watch-and-wait groups. SNOT– total sino-nasal outcome test score

The mean total SNOT score was 24.8 (SD 19, range 0–84). There was no significant difference in SNOT score between the conservative and operative groups (22.2 (SD 18) vs. 26.8 (SD 21), *p* = 0.47). There was no correlation between time since surgery and total SNOT score (*p* = 0.46). Two outliers in the postoperative group had very high SNOT scores of 83 and 82. In both cases, the surgical procedures were performed more than 2 years before the examination, so no association between the surgery and the high SNOT score could be established. The proportions of patients within each SNOT category remained similar (Fig. 3). However, the SNOT score correlated significantly with the DT, HADS-A, and HADS-D scores (Table 3), indicating an association with higher psychological distress.

**Table 3:** Correlation between scores in study cohort (Spearman’s rho)

	SNOT	HADS-A	HADS-D	DT
SNOT		0.759	0.676	0.536
HADS-A	0.759		0.747	0.603
HADS-D	0.676	0.747		0.553
DT	0.536	0.603	0.553	

*all P values < 0.001

### Identification of patients at risk

We aimed to identify patients at risk of psychological distress. A univariate logistic regression analysis was conducted using putative risk factors. Patients with DT scores ≥ 6, HADS-A, and HADS-D scores ≥ 11 were considered distressed. The SNOT test sum score was the only factor significantly associated with distress in all three scales (Table 4). Neither the time since diagnosis, treatment, nor tumor progression were significantly associated with distress.

**Table 4:** Univariate logistic regression analysis for the clinically significant distress scores. OR– odds ratio, 95%CI– confidence interval, HADS-D Depression score, HADS-A anxiety score, DT Distress thermometer score, SNOT– total sino-nasal outcome test score. The asterisk marks statistically significant values

Variable	OR (95%CI)		
	DT ≥ 6	HADS-A ≥ 11	HADS-D ≥ 11
Gender	1.35 (0.48–3.78)	1.32 (0.46–3.81)	0.80 (0.25–2.60)
Age ≥ 65 years	0.90 (0.30–2.73)	0.97 (0.31–3.01)	0.36 (0.11–1.22)
Single vs. partnership	2.38 (0.66–8.56)	1.02 (0.28–3.66)	2.64 (0.65–10.73)
Pituitary insufficiency (multiple axes)	1.36 (0.43–4.33)	0.40 (0.12–1.35)	0.55 (0.19–1.66)
Cortisone Therapy	0.71 (0.23–2.15)	1.10 (0.36–3.38)	1.33 (0.36–4.88)
Cabergoline (or other)	1.42 (0.47–4.31)	1.10 (0.36–3.38)	0.88 (0.25–3.04)
SNOT*	**1.04 (1.01–1.07)** *P* = 0.02	**1.12 (1.06–1.19)** *P* < 0.001	**1.05 (1.01–1.08)** *P* = 0.01
Operative vs. conservative treatment	0.49 (0.17–1.37)	0.93 (0.33–2.63)	0.51 (0.16–1.68)
Expansive tumor growth	1.36 (0.47–3.91)	0.89 (0.30–2.65)	1.03 (0.30–3.56)
Tumor size (micro vs. macro)	1.63 (0.48–5.50)	1.02 (0.28–3.65)	0.53 (0.10–2.80)
Time since diagnosis	1.00 (0.99–1.01)	0.99 (0.98-1.00)	1.00 (0.99–1.01)
Time since treatment	1.00 (0.98–1.03)	1.00 (0.98–1.03)	1.00 (0.97–1.03)
Tumor progress	1.49 (0.34–6.67)	1.92 (0.43–9.09)	1.27 (0.22–7.14)
Tumor regression	0.65 (0.11–3.85)	0.29 (0.03–2.63)	-

**Fig. 4:**
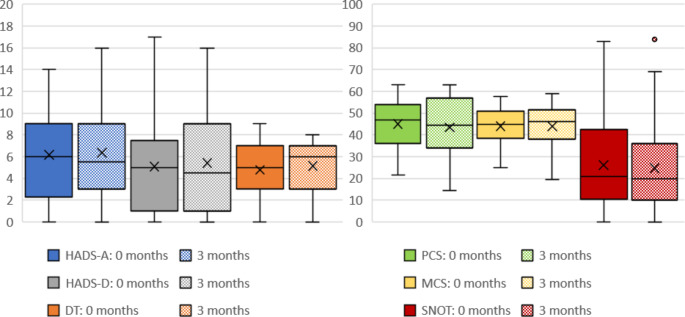
Development of mean score of applied questionnaires over time. HADS-A– anxiety score, HADS-D– depression, DT– Distress Thermometer, PCS– physical component summary, MCS– mental component summary, SNOT– total Sino-Nasal Outcome Test score

In total, 45 patients (72.6%) completed the follow-up survey. In comparison to the first survey, there were no significant differences concerning age, sex and group attribution. We did not find significant differences in mean HADS, DT, SF-36, or SNOT scores during this period in comparison to the first survey conducted in the outpatient department (Fig. 4).

## Discussion

This study confirms that there is no significant difference in self-reported distress, anxiety, depression, or quality of life between patients with operatively and conservatively managed sellar masses. The results of the 3-6-month follow-up survey further support this conclusion.

In our cohort, the mean DT score was 4.8 (SD 2.7), which is higher than in the German general population sample (3.2 (SD 2.6) [16] but comparable to the mean score in patients with meningiomas 4.5 (SD 2.5) [20]. Similar to our findings, in a study of patients operated on inactive pituitary adenomas, elevated depression and distress scores were found [21]. Lower quality of life has been reported in patients with prolactinomas, other secreting adenomas, and non-functioning tumors [3, 35]. Interestingly, the mean DT score of patients with malignant brain tumor (glioblastoma) in patients under 65 years of age was similar at 4.9 (SD 2.6) [29]. These results are consistent with those of other studies, showing that the patients with low-grade brain tumors may experience as much psycho-oncological distress as patients with a higher-grade brain tumor [15, 26]. Our study included postoperative and conservatively managed patients as well as patients with uncertain, possibly non-neoplastic entities. Regardless of tumor entity, one of the possible explanations for increased distress may be unmet information needs, which can be addressed through improvement of patient information and education [7]. Different perspectives and expectations between patients and health care providers may provide additional distress. Patients with hormonal imbalance may develop elevated levels of distress [5]. For example, in our study, the patients with ACTH-producing adenomas exhibited elevated levels of anxiety and depression, consistent with previous studies that have shown persistent physical and mental effects, even in remission [4, 23]. However, study participants with pituitary insufficiency were well compensated and did not show signs of elevated distress. A recent study demonstrated that more than 20% of patients with Cushing disease believed they had no choices in their treatment, in contrast to only 1% of health care providers [13]. This highlights the need for routine patient education and psychooncological screening in outpatient oncological care, regardless of tumor type and aggressiveness.

Compared to the surgically and conservatively managed patients with meningiomas who were recruited for a similar survey at our department [20], patients with sellar tumors demonstrated significantly lower levels of anxiety and depression (10.0 (SD 1.9) and 11.1 (SD 1.7), *p* < 0.001 for both comparisons). Moreover, HADS-A and HADS-D values observed in the current study (6.2 (SD 4.1) and 5.1 (SD 4.2)) did not deviate from the normative values in the German population [17]. The similarities in the DT score between sellar tumors and meningiomas make it challenging to explain the differences in the level of anxiety and depression between the two entities. However, based on our analysis, we state that further research is needed to fully understand the underlying factors. Sellar tumors are commonly perceived by patients as being located in a certain isolated area, not directly adjacent to the brain, which may lead to a lower level of distress. A comparison of the emotional burden of patients with anterior skull base meningioma and pituitary adenoma revealed that the patients with pituitary adenoma tended to score higher in anxiety whereas patients with meningioma tended to score higher in depression [37].

Our study included patients early after a diagnosis of sellar tumor was made as well as those who had been exposed to the diagnosis for several years. Moreover, we could not identify any changes in self-reported distress in short-term follow-up at home, which might hypothetically have reflected the distress caused by the outpatient visit. Interestingly, no association between DT, HADS-A, or HADS-D score and time since diagnosis or last treatment was observed. Because data on pituitary masses is scarce, a study on skull base meningiomas demonstrated no difference in main aspects of quality of life 5 years of more after the last intervention in comparison to the control group [10]. In contrast, another study on meningioma patients reported relevant distress nearly a decade after operation or tumor diagnosis [22].

Among all the sociodemographic and tumor-associated factors investigated, only the total SNOT score was associated with elevated distress in DT and HADS scales. Notably, the SNOT-22 correlated significantly with DT, HADS-A, and HADS-D scores. Lauriello et al. [24] reported a correlation between SNOT and HADS-A but not HADS-D in patients with allergic rhinitis. Conversely, a study of SNOT-22 in a control population revealed an association between depression and a higher SNOT score [9]. It is a well-established fact that patients with chronic rhinosinusitis have a significantly higher incidence of depression and anxiety than healthy controls [8]. Additionally, a high SNOT score has been linked with a lower quality of life [28]. A subjective postoperative outcome after nasal surgery according to SNOT showed satisfactory results and was not different in comparison from the conservative group, except for two outliers. This demonstrates the safety of transnasal pituitary surgery and its infrequent association with postoperative symptoms. Surgical removal of the prolactinomas should be considered a viable alternative, especially given the impulse control issues under dopamine agonist therapy [6].

### Limitations

This study has some limitations. First, although it was conducted in a neurosurgical center and two outpatient endocrinological services, which provides valuable insights into the field, the sample size was calculated specifically to evaluate differences of self-reported distress between study groups; therefore, further analyses are exploratory. Patient recruitment was not consecutive at outpatient endocrinological services, which may contribute to selection bias. Our analysis did not show any significant differences between the two subgroups, patients receiving conservative treatment and patients under a wait-and-watch strategy without active treatment. However, it is important to note that the number of patients in these subgroups is relative, which precludes further subgroup analysis.

Despite these shortcomings, this study shows that patients with sellar tumors experience less distress than those with other brain tumors. However, further research is needed to determine the factors that contribute to patient distress. In the meantime, it is recommended that regular patient screening (e.g. using DT) be implemented in the outpatient department to identify patients at risk and provide appropriate counseling.

## Conclusions

In our cohort, the level of self-reported distress in patients with sellar masses was not associated with a specific treatment strategy. ACTH-producing adenomas and manifest nasal symptoms were associated with higher psychological distress. Routine screening is necessary to identify patients in need of psychooncological support.

## Data Availability

No datasets were generated or analysed during the current study.

## References

[1] Baird A, Sullivan T, Zafar S, Rock J (2003) Quality of life in patients with pituitary tumors: a preliminary study. Qual Manag Health Care 12:97–105. 10.1097/00019514-200304000-00005 12747133 10.1097/00019514-200304000-00005

[2] Bullinger M (1995) German translation and psychometric testing of the SF-36 Health Survey: preliminary results from the IQOLA project. Soc Sci Med 41:1359–1366. 10.1016/0277-9536(95)00115-N 8560303 10.1016/0277-9536(95)00115-n

[3] Castle-Kirszbaum M, Biermasz N, Kam J, Goldschlager T (2024) Quality of life in Prolactinoma: a systematic review. Pituitary 27:239–247. 10.1007/S11102-024-01392-1 38656635 10.1007/s11102-024-01392-1 PMC11150290

[4] Chen Z, Wang G, Jiang C (2019) Posttraumatic stress symptoms (PTSS) in patients with Cushing’s disease before and after surgery: a prospective study. J Clin Neurosci 66:1–6. 10.1016/J.JOCN.2019.05.059 31178305 10.1016/j.jocn.2019.05.059

[5] Crespo I, Santos A, Webb SM (2015) Quality of life in patients with hypopituitarism. Curr Opin Endocrinol Diabetes Obes 22:306–312. 10.1097/MED.0000000000000169 26103454 10.1097/MED.0000000000000169

[6] Dogansen SC, Cikrikcili U, Oruk G, Kutbay NO, Tanrikulu S, Hekimsoy Z, Hadzalic A, Gorar S, Omma T, Mert M, Akbaba G, Yalin GY, Bayram F, Ozkan M, Yarman S (2019) Dopamine Agonist-Induced Impulse Control disorders in patients with Prolactinoma: a cross-sectional Multicenter Study. J Clin Endocrinol Metab 104:2527–2534. 10.1210/JC.2018-02202 30848825 10.1210/jc.2018-02202

[7] Donegan D, Gowan T, Gruber R, Cottingham A, Flanagan M, Erickson D, Imperiale TF (2021) The need for patient-centered education among patients newly diagnosed with a Pituitary Tumor. J Endocr Soc 5:1–7. 10.1210/JENDSO/BVAB061 10.1210/jendso/bvab061 PMC8143658 34056501

[8] Erskine SE, Hopkins C, Clark A, Anari S, Robertson A, Sunkaraneni S, Wilson JA, Beezhold J, Philpott CM (2017) Chronic rhinosinusitis and mood disturbance. Rhinology 55:113–119. 10.4193/RHIN16.111 28434016 10.4193/Rhin16.111

[9] Farhood Z, Schlosser RJ, Pearse ME, Storck KA, Nguyen SA, Soler ZM (2016) Twenty-two-item sino-nasal outcome test in a control population: a cross-sectional study and systematic review. Int Forum Allergy Rhinol 6:271–277. 10.1002/ALR.21668 26610073 10.1002/alr.21668

[10] Fisher FL, Zamanipoor Najafabadi AH, van der Meer PB, Boele FW, Peerdeman SM, Peul WC, Taphoorn MJB, Dirven L, van Furth WR (2021) Long-term health-related quality of life and neurocognitive functioning after treatment in skull base meningioma patients. J Neurosurg 136:1077–1089. 10.3171/2021.4.JNS203891 34598137 10.3171/2021.4.JNS203891

[11] Gittleman H, Ostrom QT, Farah PD, Ondracek A, Chen Y, Wolinsky Y, Kruchko C, Singer J, Shettry VRK, Laws ER, Sloan AE, Selman WR, Barnholtz-Sloan JS (2014) Descriptive epidemiology of pituitary tumors in the United States, 2004–2009: clinical article. J Neurosurg 121:527–535. 10.3171/2014.5.JNS131819 24926650 10.3171/2014.5.JNS131819

[12] Goebel S, Mehdorn HM (2011) Measurement of psychological distress in patients with intracranial tumours: the NCCN distress thermometer. J Neurooncol 104:357–364. 10.1007/s11060-010-0501-5 21188470 10.1007/s11060-010-0501-5

[13] Halstrom A, Lin IH, Lin A, Cohen M, Tabar V, Geer EB (2024) Different patient versus provider perspectives on living with Cushing’s disease. Pituit doi. 10.1007/S11102-024-01381-4 10.1007/s11102-024-01381-4 PMC11009766 38315244

[14] Herrmann C, Buss U, Huber RS-B (1995) U HADS-D hospital anxiety and depression scale–Deutsche version

[15] Hickmann AK, Nadji-Ohl M, Haug M, Hopf NJ, Ganslandt O, Giese A, Renovanz M (2016) Suicidal ideation, depression, and health-related quality of life in patients with benign and malignant brain tumors: a prospective observational study in 83 patients. Acta Neurochir (Wien) 158:1669–1682. 10.1007/s00701-016-2844-y 27318813 10.1007/s00701-016-2844-y

[16] Hinz A, Mitchell AJ, Dégi CL, Mehnert-Theuerkauf A (2019) Normative values for the distress thermometer (DT) and the emotion thermometers (ET), derived from a German general population sample. Qual Life Res 28:277–282. 10.1007/S11136-018-2014-1 30284181 10.1007/s11136-018-2014-1

[17] Hinz A Research EB-J of psychosomatic, 2011 undefined normative values for the hospital anxiety and depression scale (HADS) in the general German population. Elsevier 10.1016/j.jpsychores.2011.01.005 21767686

[18] Hopkins C, Gillett S, Slack R, Lund VJ, Browne JP (2009) Psychometric validity of the 22-item Sinonasal Outcome Test. Clin Otolaryngol 34:447–454. 10.1111/J.1749-4486.2009.01995.X 19793277 10.1111/j.1749-4486.2009.01995.x

[19] Johnson MD, Woodburn CJ, Lee Vance M (2003) Quality of life in patients with a pituitary adenoma. Pituitary 6:81–87. 10.1023/B:PITU.0000004798.27230.ED 14703017 10.1023/b:pitu.0000004798.27230.ed

[20] Kalasauskas D, Keric N, Abu Ajaj S, von Cube L, Ringel F, Renovanz M (2020) Psychological Burden in Meningioma patients under a wait-and-watch strategy and after complete resection is high-results of a prospective single Center Study. Cancers (Basel) 12:1–13. 10.3390/cancers12123503 10.3390/cancers12123503 PMC7761113 33255551

[21] Karppinen A, Ritvonen E, Roine R, Sintonen H, Vehkavaara S, Kivipelto L, Grossman AB, Niemelä M, Schalin-Jäntti C (2016) Health-related quality of life in patients treated for nonfunctioning pituitary adenomas during the years 2000–2010. Clin Endocrinol (Oxf) 84:532–539. 10.1111/CEN.12967 26493182 10.1111/cen.12967

[22] Keshwara SM, Gillespie CS, Mustafa MA, George AM, Richardson GE, Clynch AL, Wang JZ, Lawson DDA, Gilkes CE, Farah JO, Yousaf J, Chavredakis E, Mills SJ, Brodbelt AR, Zadeh G, Millward CP, Islim AI, Jenkinson MD (2023) Quality of life outcomes in incidental and operated meningiomas (QUALMS): a cross-sectional cohort study. J Neurooncol 161:317. 10.1007/S11060-022-04198-Y 36525165 10.1007/s11060-022-04198-y PMC9756745

[23] Van Der Klaauw AA, Kars M, Biermasz NR, Roelfsema F, Dekkers OM, Corssmit EP, Van Aken MO, Havekes B, Pereira AM, Pijl H, Smit JW, Romijn JA (2008) Disease-specific impairments in quality of life during long-term follow-up of patients with different pituitary adenomas. Clin Endocrinol (Oxf) 69:775–784. 10.1111/J.1365-2265.2008.03288.X 18462264 10.1111/j.1365-2265.2008.03288.x

[24] Lauriello M, Rubbo V, Di, Sinatti G, Pasqua M, Tucci C, Marco G-P, di, Necozione S, Eibenstein A (2019) Correlation between SNOT-22, nasal cytology, and Mood disorders in patients with allergic Rhinitis treated with a liposomal nasal spray. Allergy Rhinol 10:215265671986680. 10.1177/2152656719866809 10.1177/2152656719866809 PMC6676260 31413887

[25] Low JT, Ostrom QT, Cioffi G, Neff C, Waite KA, Kruchko C, Barnholtz-Sloan JS (2022) Primary brain and other central nervous system tumors in the United States (2014–2018): a summary of the CBTRUS statistical report for clinicians. Neuro-Oncology Pract 9:165. 10.1093/NOP/NPAC015 10.1093/nop/npac015 PMC9113389 35601966

[26] Mayer S, Fuchs S, Fink M, Schäffeler N, Zipfel S, Geiser F, Reichmann H, Falkenburger B, Skardelly M, Teufel M (2021) Hope and Distress are not Associated with the Brain Tumor Stage. Front Psychol 12. 10.3389/FPSYG.2021.642345 10.3389/fpsyg.2021.642345 PMC8192812 34122231

[27] Milian M, Honegger J, Gerlach C, Psaras T (2013) Health-related quality of life and psychiatric symptoms improve effectively within a short time in patients surgically treated for pituitary tumors–a longitudinal study of 106 patients. Acta Neurochir (Wien) 155:1637–1645. 10.1007/S00701-013-1809-7 23836354 10.1007/s00701-013-1809-7

[28] Philpott C, Erskine S, Hopkins C, Coombes E, Kara N, Sunkareneni V, Anari S, Salam M, Farboud A, Clark A (2016) A case-control study of medical, psychological and socio-economic factors influencing the severity of chronic rhinosinusitis. Rhinology 54:134–140. 10.4193/RHINO15.272 27172454 10.4193/Rhino15.272

[29] Renovanz M, Hickmann AK, Nadji-Ohl M, Keric N, Weimann E, Wirtz CR, Singer S, Ringel F, Coburger J (2020) Health-related quality of life and distress in elderly vs. younger patients with high-grade glioma—results of a multicenter study. Support Care Cancer 28:5165–5175. 10.1007/s00520-020-05354-8 32060706 10.1007/s00520-020-05354-8 PMC7546979

[30] Riedl D, Dejaco D, Steinbichler TB, Innerhofer V, Gottfried T, Bektic-Tadic L, Giotakis AI, Rumpold G, Riechelmann H (2021) Assessment of health-related quality-of-life in patients with chronic Rhinosinusitis - Validation of the German sino-nasal outcome Test-22 (German-SNOT-22). J Psychosom Res 140. 10.1016/J.JPSYCHORES.2020.110316 10.1016/j.jpsychores.2020.110316 33271403

[31] Sarris CE, Little AS, Kshettry VR, Rosen MR, Rehl RM, Haegen TW, Rabinowitz MR, Nyquist GG, Recinos PF, Sindwani R, Woodard TD, Farrell CJ, Santarelli GD, Milligan J, Evans JJ (2021) Assessment of the validity of the Sinonasal outcomes Test-22 in pituitary surgery: a Multicenter prospective trial. Laryngoscope 131:E2757–E2763. 10.1002/LARY.29711 34196397 10.1002/lary.29711

[32] Schmidt CO, Hegenscheid K, Erdmann P, Kohlmann T, Langanke M, Völzke H, Puls R, Assel H, Biffar R, Grabe HJ (2013) Psychosocial consequences and severity of disclosed incidental findings from whole-body MRI in a general population study. Eur Radiol 23:1343–1351. 10.1007/S00330-012-2723-8 23239059 10.1007/s00330-012-2723-8

[33] Teramoto A, Hirakawa K, Sanno N, Osamura Y (1994) Incidental pituitary lesions in 1,000 unselected autopsy specimens. Radiology 193:161–164. 10.1148/RADIOLOGY.193.1.8090885 8090885 10.1148/radiology.193.1.8090885

[34] Toma S, Hopkins C (2016) Stratification of SNOT-22 scores into mild, moderate or severe and relationship with other subjective instruments. Rhinology 54:129–133. 10.4193/RHINO15.072 27017484 10.4193/Rhino15.072

[35] Vega-Beyhart A, Enriquez-Estrada VM, Bello-Chavolla OY, Torres-Victoria TR, Martínez-Sánchez FD, López-Navarro JM, Pérez-Guzmán MC, Hinojosa-Amaya JM, León-Suárez A, Espinoza-Salazar HD, Roldán-Sarmiento P, Gómez-Sámano MA, Gómez-Pérez FJ, Cuevas-Ramos D (2019) Quality of life is significantly impaired in both secretory and non-functioning pituitary adenomas. Clin Endocrinol (Oxf) 90:457–467. 10.1111/CEN.13915 30548674 10.1111/cen.13915

[36] Vernooij MW, Ikram MA, Tanghe HL, Vincent AJPE, Hofman A, Krestin GP, Niessen WJ, Breteler MMB, Van Der Lugt A (2007) Incidental findings on brain MRI in the general population. N Engl J Med 357:1821–1828. 10.1056/NEJMoa070972 17978290 10.1056/NEJMoa070972

[37] Wagner A, Shiban Y, Kammermeier V, Joerger AK, Lange N, Ringel F, Meyer B, Shiban E (2019) Quality of life and emotional burden after transnasal and transcranial anterior skull base surgery. Acta Neurochir (Wien) 161:2527–2537. 10.1007/S00701-019-04062-5 31602535 10.1007/s00701-019-04062-5

[38] Ware JE, Sherbourne CD (1992) The MOS 36-item short-form health survey (Sf-36): I. conceptual framework and item selection. Med Care 30:473–483. 10.1097/00005650-199206000-00002 1593914

[39] Zhang Y, Guo X, Wang L, Guo J, Zhao H, Sun S, Sun Y, Xu D, Wang Z, Gao L, Feng M, Xing B (2020) Pre- and Postoperative Health Status of patients with nonfunctioning and secretory pituitary adenomas and an analysis of related factors. Int J Endocrinol 2020. 10.1155/2020/4056591 10.1155/2020/4056591 PMC7189308 32377185

[40] Zigmond AS, Snaith RP (1983) The Hospital anxiety and Depression Scale. Acta Psychiatr Scand 67:361–370. 10.1111/j.1600-0447.1983.tb09716.x 6880820 10.1111/j.1600-0447.1983.tb09716.x

